# Are We Reaching the Limits of *Homo sapiens*?

**DOI:** 10.3389/fphys.2017.00812

**Published:** 2017-10-24

**Authors:** Adrien Marck, Juliana Antero, Geoffroy Berthelot, Guillaume Saulière, Jean-Marc Jancovici, Valérie Masson-Delmotte, Gilles Boeuf, Michael Spedding, Éric Le Bourg, Jean-François Toussaint

**Affiliations:** ^1^Institut de Recherche bio-Médicale et d'Epidémiologie du Sport (IRMES) EA 7329, Institut National du Sport, de l'Expertise et de la Performance, Université Paris Descartes, Université Sorbonne Paris Cité, Paris, France; ^2^Laboratoire Matière et Systèmes Complexes, UMR 7057 Université Paris Diderot, Centre National de la Recherche Scientifique, Université Sorbonne Paris Cité, Paris, France; ^3^Group Adaptation and Prospective, High Council of Public Health, Paris, France; ^4^Research Laboratory for Interdisciplinary Studies, Paris, France; ^5^Carbone 4, Paris, France; ^6^Laboratoire des Sciences du Climat et l'Environnement, Institut Pierre Simon Laplace, CEA-Centre National de la Recherche Scientifique, Université de Versailles Saint-Quentin en Yvelines, Gif-sur-Yvette, France; ^7^Muséum National d'Histoire Naturelle, Université Pierre et Marie Curie, Sorbonne Universités, Paris, France; ^8^IUPHAR and Spedding Research Solutions SAS, Le Vésinet, France; ^9^Centre de Recherches sur la Cognition Animale, Centre de Biologie Intégrative, Université de Toulouse, Centre National de la Recherche Scientifique, Université Toulouse III Paul Sabatier, Toulouse, France; ^10^Centre d'Investigations en Médecine du Sport (CIMS), Hôtel-Dieu de Paris, Assistance Publique - Hôpitaux de Paris, Paris, France

**Keywords:** anthropocene, human upper limits, performance, longevity, life span, biometry, public health, environment

## Abstract

Echoing scientific and industrial progress, the Twentieth century was an unprecedented period of improvement for human capabilities and performances, with a significant increase in lifespan, adult height, and maximal physiological performance. Analyses of historical data show a major slow down occurring in the most recent years. This triggered large and passionate debates in the academic scene within multiple disciplines; as such an observation could be interpreted as our upper biological limits. Such a new phase of human history may be related to structural and functional limits determined by long term evolutionary constraints, and the interaction between complex systems and their environment. In this interdisciplinary approach, we call into question the validity of subsequent forecasts and projections through innovative and related biomarkers such as sport, lifespan, and height indicators. We set a theoretical framework based on biological and environmental relevance rather than using a typical single-variable forecasting approach. As demonstrated within the article, these new views will have major social, economical, and political implications.

## Introduction

How long can we live (Olshansky et al., 1990, 2001; Aarssen and de Haan, 1994; Wilmoth, 1998; Thatcher, 1999; Oeppen and Vaupel, 2002; Watts et al., 2006; Carnes and Olshansky, 2007; Hayflick, 2007; Christensen et al., 2009; Olshansky and Carnes, 2009, 2013; Weon and Je, 2009; Vaupel, 2010; Couzin-Frankel, 2011; Li et al., 2011; Beltrán-Sánchez et al., 2012; Bravo and Real, 2012; Le Bourg, 2012; da Silva Antero-Jacquemin et al., 2014; Finch et al., 2014; Dong et al., 2016; Hanayama and Sibuya, 2016; Brown et al., 2017; de Beer et al., 2017; Gavrilov et al., 2017; Gbari et al., 2017; Hughes and Hekimi, 2017; Kirkwood, 2017; Le Bourg and Vijg, 2017; Lenart and Vaupel, 2017; Rootzén and Zholud, 2017; Rozing et al., 2017; Vijg and Le Bourg, 2017)? How fast can we run or swim (Kennelly, 1906; Hill, 1925; Deakin, 1967; Chatterjee and Chatterjee, 1982; Whipp and Ward, 1992; Blest, 1996; Reinboud, 2004; Tatem et al., 2004; Nevill and Whyte, 2005; Kuper and Sterken, 2007; Nevill et al., 2007; Berthelot et al., 2008, 2010b, 2015; Denny, 2008; Desgorces et al., 2008, 2012; Lippi et al., 2008; Weyand et al., 2010; Liu et al., 2012; Haake et al., 2014; Haugen et al., 2015; Kinugasa and Usami, 2016)? Demographers disagree about the lifespan trend and its potential limit, while sports scientists discuss the frontiers of maximal physical performance. Such questions stimulate large and passionate debates about the potential of *Homo sapiens* and its biological upper limits. Historical series, defined as the measurable data collected since the nineteenth century for lifespan, sport, or height provide crucial information to understand human physiology and the form and nature of our progression over the last 10 generations.

Recent studies about lifespan trends (da Silva Antero-Jacquemin et al., 2014; Dong et al., 2016; Hughes and Hekimi, 2017) increased interest about the possible ceilings in longevity for humans. This long-lasting debate increased in strength at the beginning of the 1990s (Olshansky et al., 1990; Oeppen and Vaupel, 2002). Using biological and evolutionary arguments, the first leading opinion postulated an upper limit for life expectancy at birth and maximal longevity (Carnes and Olshansky, 2007; Hayflick, 2007; Olshansky and Carnes, 2009, 2013; Le Bourg, 2012). These limits may have already been approached: around 85–95 years for life-expectancy and 115–125 years for maximal longevity, as a result of nutritional, medical, societal, and technological progress (Carnes and Olshansky, 2007; Olshansky and Carnes, 2009; da Silva Antero-Jacquemin et al., 2014; Dong et al., 2016). A second school of thought considered that life expectancy may continue to progress indefinitely at a pace of 2 to 3 added years per decade (Oeppen and Vaupel, 2002; Christensen et al., 2009; Vaupel, 2010). They claim that most of the babies born during the 2000s, “if the present yearly growth in life expectancy continues through the twenty-first century,” will celebrate their 100th birthday (Christensen et al., 2009) or, potentially reach physical immortality due to undefined scientific breakthroughs (de Grey, 2003; Kurzweil and Grossman, 2010).

Analyzing historical trends in sports series, a similar debate took place about physical performance upper limits (Kennelly, 1906; Hill, 1925; Whipp and Ward, 1992; Blest, 1996; Kuper and Sterken, 2003, 2007; Tatem et al., 2004; Nevill and Whyte, 2005; Nevill et al., 2007; Berthelot et al., 2008, 2010b, 2015; Denny, 2008). Since the former estimations of Kennelly (1906) or the pioneering work of Hill (1925), several studies have forecast future achievements (Blest, 1996; Nevill and Whyte, 2005; Nevill et al., 2007; Berthelot et al., 2008, 2010b, 2015). In one of the most extreme cases, Tatem and colleagues asserted in 2004 that performances progress linearly. They proposed that women would outrun men by 2156 in the 100 m track event (Tatem et al., 2004). A few studies also suggested continuous progression of records within upcoming decades or centuries (Whipp and Ward, 1992; Kinugasa and Usami, 2016; Rozing et al., 2017). Other authors considered that an upper limit gradually appears (Reinboud, 2004; Nevill and Whyte, 2005; Nevill et al., 2007; Berthelot et al., 2008, 2010a, 2015).

However, beyond mathematical projections and personal beliefs, these debates rarely integrate biological and environmental aspects of such trends (Carnes et al., 2014; Finch et al., 2014). Here, we will put such parameters into perspective as they shape common trends in lifespan, physical performance and height data. First, we will propose an overview of the body design and physical limits, as previously described in the longevity debate (Carnes and Olshansky, 2007; Olshansky and Carnes, 2009; Le Bourg, 2012), the structural and functional delimitations of the human organism integrating specific biological constraints resulting from evolutionary and environmental constraints. In light of these delimitations, we will then provide an analysis of the simultaneous progression of biometrical indicators under favorable environments. Finally, we will discuss the perspectives derived from the recently observed plateaus, which suggest upper biological limits for *H. sapiens* or an increase of its environmental constraints.

## Limited capabilities

### Physically and functionally delimited organism

Most species share common features, including Mendelian inheritance (Dhar and Giuliani, 2010), individual growth and decline (e.g., the age-related changes in physical and cognitive performances) (Moore, 1975; Berthelot et al., 2011; Marck et al., 2016) and allometric scaling for energetic relationships (e.g., metabolic rate scales as the 3/4-power of mass) (Speakman, 2005; West and Brown, 2005). Following these emerging properties, individuals from our *H. sapiens* species are a delimited organism shaped by evolutionary constraints and modulated by their interactions with the environment.

The human body, as in any living species, is a finite organism, with structural (640 muscles and 206 bones) and functional boundaries at every level of organization. Each cell embeds around 20 to 25,000 protein-encoding genes over 2.85 billion nucleotides aligned among 46 chromosomes, shaping the human organism during development (Ezkurdia et al., 2014). From one cell at fecundation to ~3 × 10^13^ cells classified into more than 300 different types, the human organism constitutes a precisely delimited body (Bianconi et al., 2013).

Cells have a limited replicative potential depending on their type (Hayflick, 1965; Campisi, 1996). In addition, they accumulate damage with aging and replication, causing dysfunctions, while apoptotic and necrotic processes contribute to their gradual loss (Campisi, 1996). Such a process is common in muscle tissues that gradually lose fibers during aging, or among neurons or hematopoietic stem cells, further aggravating their functional decline (Mitchell et al., 2012; Holstege et al., 2014; Marck et al., 2016).

Each adult organ possesses capabilities and a particular size that is species-specific, with some variability (Poole and Erickson, 2011). For example, the normal human resting heart rate varies between 45 and 100 beats per minute (bpm), reaching a maximum of 220 bpm during intensive effort (Tanaka et al., 2001; Mason et al., 2007; Nes et al., 2013). This maximal rate, which depends on age (Tanaka et al., 2001), is the upper functional performance of a normal human heart. Each organ has a similar age-dependent potential (Bassett and Howley, 2000); the maximal functional value usually reaches a peak during the third decade of life, and then gradually declines (Schoenberg et al., 1978; Wiswell et al., 2001; Mitchell et al., 2012; Kusy and Zielinski, 2014; Marck et al., 2016).

Such limitations of the human body and alterations with age are a subject of debate (de Grey, 2003; Carnes and Olshansky, 2007; Olshansky and Carnes, 2009; Kurzweil and Grossman, 2010). Prolongevists claim that aging could be delayed, slowed, reversed, or even eradicated during the next decades (de Grey, 2003; Carnes and Olshansky, 2007; Olshansky and Carnes, 2009; Kurzweil and Grossman, 2010; Vaupel, 2010). In sport, similar ideas suggest enhancement of the body's design, (e.g., by using gene therapy) (Berthelot et al., 2015). However, despite boisterous announcements, recent research has yet to deliver even one element showing how to enhance any human maximal performance.

### Long and short term limitations through evolutionary and ecosystemic constraints

Recent human biometrical progression resulted from both long term human evolution and recent societal changes. It has been proposed that human evolution toward bipedalism and running may have paralleled climatic changes in Africa during the last three million years (Ruff, 1991; Bramble and Lieberman, 2004; Noakes and Spedding, 2012). It was associated with skeletal alterations and increased metabolic capacity as compared with non-human primates (Bramble and Lieberman, 2004; Lieberman and Bramble, 2007; Noakes and Spedding, 2012). At the same time, humans developed a large brain with spatial and social memory characteristics, which also resulted from a long adolescence (Neubauer and Hublin, 2012; Hublin et al., 2015). Changes in diet and more frequent meat-eating were critical (Domínguez-Rodrigo et al., 2010; Zaatari et al., 2016). A common driving force may have participated in the growth of both running and cognitive functions (Noakes and Spedding, 2012). Skeletal changes allowing high-speed throwing, important during hunting, also evolved ~2 million years ago (Roach et al., 2013). These changes form the background for many modern sports and physiological limits which have been derived from this evolutionary period.

Modern humans ventured out of Africa to occupy almost all terrestrial niches (Nielsen et al., 2017). Both biology and societies evolved and allowed humans to adapt to the most extreme surroundings. Recently, the industrial revolution deeply changed our environment progressively reducing its constraints on our daily habits, simultaneously triggering a global transition of living conditions and health in less than 10 generations (Wilmoth, 2000; Fogel, 2004; Omran, 2005; Steffen et al., 2007, 2011; McMichael, 2014). This was supported by a much higher primary energy consumption *per capita* (raised by one to two orders) and had large epidemiological (infections replaced by degenerative diseases as the main mortality cause), nutritional (less cereals, more meat, fat, and sugar in daily diets), agricultural (ten times higher yields), and demographical consequences (a mortality decrease followed by birth rate reduction a few decades later, allowing for the natural population increase) (Wilmoth, 2000; Fogel, 2004; Omran, 2005; Steffen et al., 2007, 2011; McMichael, 2014).

During the nineteenth and twentieth centuries, human beings grew taller and lived longer (Wilmoth, 2000; Fogel, 2004; Olshansky and Carnes, 2009; Le Bourg, 2012; NCD-RisC, 2016). Life span and height echo development in many countries; their growth rates have been associated with energetic, nutritional, scientific, medical, and industrial progresses (Fogel, 2004; Omran, 2005; Le Bourg, 2012; Carnes et al., 2014; Finch et al., 2014; NCD-RisC, 2016; Stulp and Barrett, 2016). Likewise, metric or chronometric measurements of maximal physiological performances document such progress since the first modern Olympic Games in 1896. This allows for the development of accurate indicators and tools measuring human progression rates (Hill, 1925; da Silva Antero-Jacquemin et al., 2014; Berthelot et al., 2015).

These recent changes are highly dependent on phylogenetic and demographic constraints that provide a limited number of new phenotypic variants among recent generations. These generations inherit specific capabilities from the previous ones, limiting possible adaptations (Blomberg and Garland, 2002). On another timescale, physical and chemical constraints such as gravity or osmotic properties restrain the possible organism variations, most of them since the beginning of terrestrial life. As such, modern human potential, including an enlarged brain, height, lifespan, and physical performance has been dependent on very long-term evolutionary parameters (Kirkwood and Austad, 2000; Le Bourg, 2001; Bramble and Lieberman, 2004; Noakes and Spedding, 2012; Stulp and Barrett, 2016; Kirkwood, 2017; Le Bourg and Vijg, 2017; Vijg and Le Bourg, 2017). These constraints, applied to a singular genotype, shape a delimited organism in the course of its development, whose traits vary according to the possibilities of the species's genome. With such variability human height, for example, shows a one to five span (in centimeters) between the smallest and tallest adult individuals (from 55 to 272 cm, both situations usually induce severe pathological conditions and major health risks) (Stulp and Barrett, 2016). However, these extreme phenotypes can be considered as the present lower and upper known limits of the potential height for *H. sapiens*.

In addition, population size is another factor that influences the progression of maximal biometrical values (Gillespie, 2001; Charlesworth, 2009; Foster et al., 2010; Lanfear et al., 2014). As the size of the population increases, the tails of the indicator distributions expand allowing extremely rare phenotypes, such as Jeanne Calment for longevity or Usain Bolt for maximal running speed (Williams and Folland, 2008).

Phenotypic plasticity depends on the environmental conditions; it is important in shaping both phenotypic changes in response to ecological alterations and ecological changes in response to phenotypic adaptation (DeWitt et al., 1998; Merilä and Hendry, 2014; Stulp and Barrett, 2016). Recent adult height evolution is an example of such plasticity, which primarily depended on nutrition availability, hygiene, and healthcare throughout infancy (NCD-RisC, 2016; Stulp and Barrett, 2016). In the last century, technological developments and their accelerated diffusion allowed for rapid phenotypic changes, with a major improvement in most physiological factors among humans and human-driven species (Fogel, 2004; Omran, 2005; Berthelot et al., 2015). This period covers a typical expansion of major physiological indicators that could be interpreted as a non-genetic “techno-physiological” evolution (Fogel, 2004) with a large phenotypic expansion (Berthelot et al., 2011) (epigenetic changes being more likely expected on such a brief time scale (Gapp et al., 2014; Bohacek and Mansuy, 2015; Fumagalli et al., 2015). This evolution was associated with a large increase of the energy available *per capita* (Fogel, 2004) and supported by a synergy between economic growth, medical advancements and technological diffusion inducing a positive feedback in the efficiency of energy production and use (Fogel, 2004; Steffen et al., 2007, 2011; Carnes et al., 2014; Finch et al., 2014; McMichael, 2014).

However, mankind is now the major actor implicated in its own environment alterations (Steffen et al., 2007, 2011; IPCC, 2014; McMichael, 2014). *Sapiens* alters his ecosystem, while the ecosystem also shapes him in return (Steffen et al., 2011). Our activities have been implicated as the dominant cause of most environment changes and the recent acceleration could have major impacts on human health, even if some progress has been recently made, such as the increased use of renewable energy (Steffen et al., 2007, 2011; IPCC, 2014; McMichael, 2014).

Physiological traits are directly and indirectly affected by environmental changes. For example, temperature plays a crucial role in mortality rates, in sport performances or in the interactions between species (Laaidi et al., 2006; El Helou et al., 2012; Grigaltchik et al., 2012; Haïda et al., 2013; Carnes et al., 2014; Finch et al., 2014; Berthelot et al., 2015). In running, best performances describe an inverted-U shaped curve, with an optimal temperature at about 10°C for marathon and 23°C for sprint distances (100 m) (El Helou et al., 2012; Haïda et al., 2013; Berthelot et al., 2015). A similar relation links temperature and survival rates, with an optimum value between 20 and 26°C (Laaidi et al., 2006). Major temperature elevations during current climate changes may have unfavorable impacts on our capacity to reach our functional maxima and absolute physical limits.

Finally, several sectors also show signs of saturation, including agriculture under the pressure of large demographic growth, demonstrating crop yield stagnation (IEA, 2016), soil overexploitation, and perturbations of ecological processes leading to large losses in biodiversity (Cardinale et al., 2012; Maxwell et al., 2016). Regarding the complex interactions between organisms and their milieu, enhanced environmental constraints and reduced primary resources at the same time may be the simultaneous keys for predicting future human performances (Rockström et al., 2009; Carnes et al., 2014; Finch et al., 2014).

## To the upper limits

### Physical performances

“*There is a vast store of accurate information, hitherto almost unexploited, in the records of athletic sports and racing*” (Hill, 1925). Since the pioneering work of A.V. Hill, sport performances have provided valuable data to grasp human physiology, as they represent the most accurate measurements of human potentials and capabilities (Hill, 1925; Larry et al., 2012; Hawley et al., 2014; Berthelot et al., 2015). Their historical trends provide precise ways of measuring human progress, its consecutive steps and determinants.

As no one has been able to surpass their performance, record holding athletes express the limits of human physiology (Hill, 1925; Norton and Olds, 2001; Berthelot et al., 2015). Yearly maximal performances also represent a unique biomarker for the understanding of age-related changes across lifespan at both individual and population levels, providing a simple phenotypic indicator of growth and aging (Berthelot et al., 2011; Larry et al., 2012; Justice et al., 2016; Marck et al., 2016). Finally, maximal performance also appears as a main indicator of the relationship linking physiology and health to environmental changes (for example, the impacts of temperature changes) (El Helou et al., 2012; Larry et al., 2012; Haïda et al., 2013).

**Figure 1:**
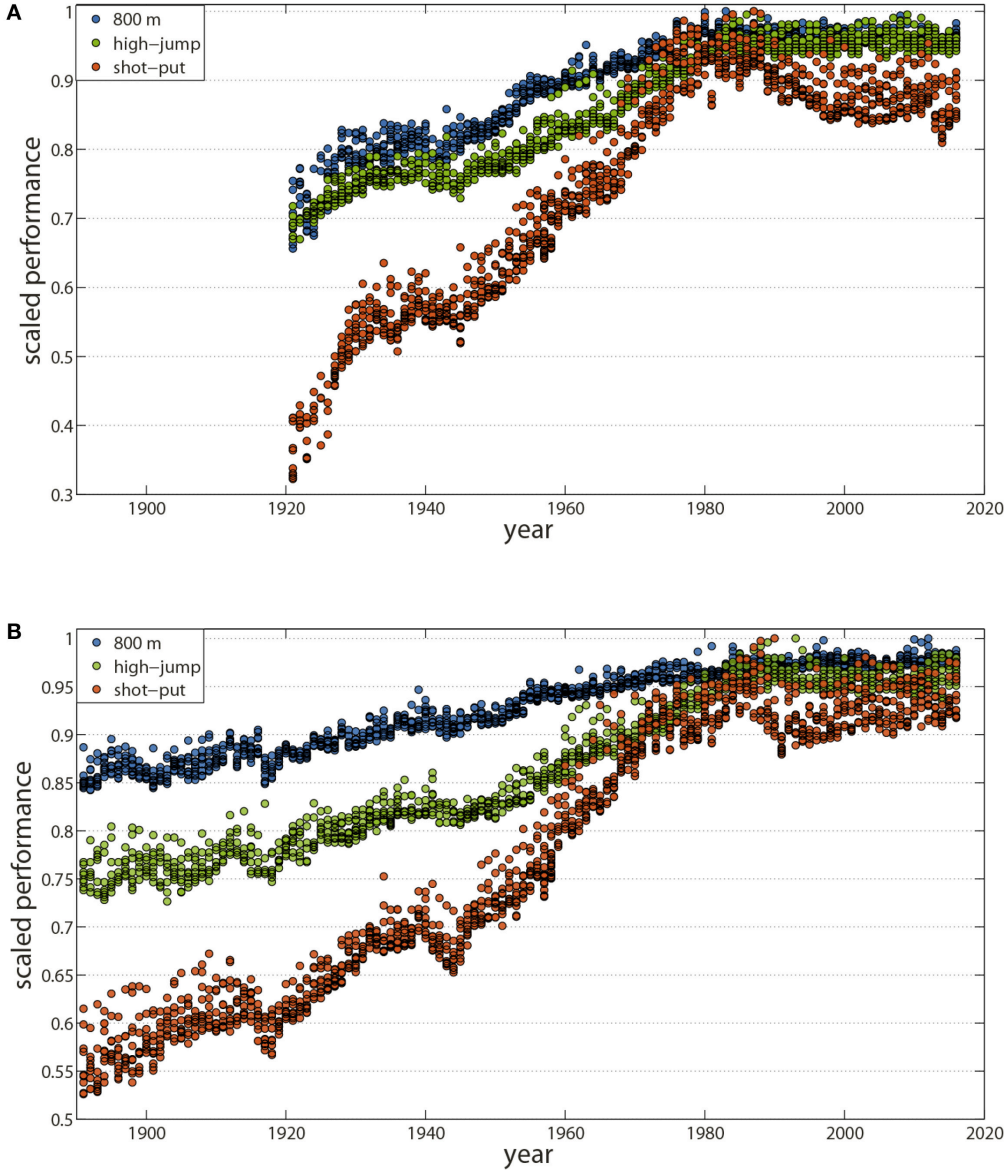
Maximal physical performance has plateaued during the last three decades. Historical series of the best performances in Track and Field events have shown trends of plateauing since 1980 for both men and women, one of the early signs of established human physiological limits. **(A)** Annual World's 10 best performances from 1921 to 2016 in women for the 800 m event (blue), the high-jump event (green), and the shot-put (orange). **(B)** Annual World's 10 best performances from 1896 to 2016 in men for the 800 m event (blue), the high-jump event (green), and the shot-put (orange). Data are from International Association of Athletics Federations (IAAF: https://www.iaaf.org/home) and are normalized by the respective world records for the event at the end of 2016, that is, performances are divided by the current world record.

The analysis of historical sport achievements offers an accurate and original reading of our society's progressions and accelerations during the twentieth century (Guillaume et al., 2009). Following the Olympic motto “*citius, altius, fortius*,” best performances in all metric and chronometric sports including running (Nevill and Whyte, 2005; Berthelot et al., 2008, 2010b, 2015; Denny, 2008), swimming (Nevill et al., 2007; Berthelot et al., 2008, 2010a, 2015), jumping (Berthelot et al., 2008, 2010a, 2015), weightlifting (Berthelot et al., 2008), cycling (Desgorces et al., 2008; El Helou et al., 2010), skiing (Desgorces et al., 2008), or skating (Kuper and Sterken, 2003; Berthelot et al., 2008) considerably progressed, until the end of the twentieth century, except during World Wars I and II (Figures 1A,B).

In the marathon, the best time by men decreased from the first world best performance ratified by the IAAF (International Association of Athletics Federations) in 1908 (2 h 55 min 18 s) to the current world best performance (2 h 02 min 57 s). Likewise, in the 100 m swimming free style the world record progressed from 65 s in 1905 to 49.91 s in 2009, while the hour cycling record increased from 26.508 km in 1876 to 54.526 km in 2015.

Can we forecast future world record evolution in sports? Recent data have shown a common slow-down in both frequency and increments for all the Olympic chronometric and metric events (Berthelot et al., 2008, 2010b). Historical series from 1896 to 2016 in track and field, swimming, cycling, skating, and weight-lifting reveal a similar pattern suggesting a trend toward a plateau during the last three decades for both sexes (Blest, 1996; Reinboud, 2004; Berthelot et al., 2008, 2010b, 2015; Denny, 2008; Haugen et al., 2015) (Figures 1A,B). In fact, despite an average lag of 30 years between the beginning of competition for men and women, performances by women also began to plateau since the mid-1980s (Thibault et al., 2010; Berthelot et al., 2015).

In the absence of future changes in rules or technological improvements, plateaus obtained after an asymptotic progression may now indicate the potential upper limits of *H. sapiens*. This was suggested by former biomechanical and physiological investigations including structural and functional factors such as limiting oxygen uptake, maximal heart rate, muscle mass and contraction, reaction time, stature, and stride length and frequency (Bennett, 1989; Bassett and Howley, 2000; Weyand et al., 2010; Ferretti et al., 2011; Berthelot et al., 2015; Haugen et al., 2015). Moreover, recent trends in track and field (e.g., regression in Throws and Jumps) suggest that limits have already been artificially enhanced through doping practices (Spedding and Spedding, 2008; Berthelot et al., 2015). Pharmacological innovations and the Cold War exacerbated the use of performance-enhancing drugs including EPOs, growth hormones, steroid hormones, or amphetamines (Franke and Berendonk, 1997; Spedding and Spedding, 2008; Guillaume et al., 2009; Berthelot et al., 2010a, 2015). Their effects on elite athletes are difficult to precisely measure, but are certainly related to the last “burst” of performance during the eighties and nineties (Spedding and Spedding, 2008; Berthelot et al., 2010a, 2015; El Helou et al., 2010; Durussel et al., 2013). Also, technology is a main source of potential enhancement. The three successive generation of suits over the 1990–2009 period improved world swimmers' performance by a mean of 3% before their ban in 2010 (Berthelot et al., 2010a, 2015).

In the general population, long historical series in Western Europe or Scandinavian countries shows a decline in endurance and strength performance of young men (Rasmussen et al., 1999; Santtila et al., 2006; Tomkinson, 2007; Huotari et al., 2010; Runhaar et al., 2010). Studies indicate that fewer individuals reach excellent physical performance, while more and more subjects remain at low physical performance levels (Santtila et al., 2006; Huotari et al., 2010). Regression in fitness capacity may be due to reduced physical activity in most developed societies (Tomkinson, 2007; Tomkinson and Olds, 2007; Tomkinson et al., 2012; Lang et al., 2016).

Time series in sports are certainly one of the most relevant evidences in the understanding of human upper limits. They not only assess the synergistic process of human improvement until its full optimization, but also relate to the finite body of each athlete with her or his absolute limits at each spatial and temporal scale.

### Lifespan

Human life-expectancy and maximal lifespan trends also provide long historical series (Olshansky et al., 1990; Oeppen and Vaupel, 2002; Le Bourg, 2012; da Silva Antero-Jacquemin et al., 2014; Dong et al., 2016). Similar to sport achievements, though somewhat less precisely measured, it followed an unprecedented progression during the twentieth century supported by major nutritional, scientific, technological, societal, and medical innovations (Wilmoth, 2000; Oeppen and Vaupel, 2002; Fogel, 2004; Omran, 2005; Olshansky and Carnes, 2009; Le Bourg, 2012; da Silva Antero-Jacquemin et al., 2014). From 1900 to 2000 in the majority of high-income countries, life expectancy at birth increased by ~30 years (Wilmoth, 2000; Hayflick, 2007), mostly due to a reduction of child mortality through nutrition, hygiene, vaccination, and other medical improvements (Wilmoth, 2000; Fogel, 2004; Omran, 2005; Hayflick, 2007).

Concerning the future, trends oscillate, from pessimistic to optimistic views (Vaupel, 1997; Wilmoth, 2000; Oeppen and Vaupel, 2002; Christensen et al., 2009; Olshansky and Carnes, 2009; Le Bourg, 2012; da Silva Antero-Jacquemin et al., 2014; Dong et al., 2016; Gavrilov et al., 2017; Kirkwood, 2017; Le Bourg and Vijg, 2017; Vijg and Le Bourg, 2017), but recent data suggest a slow-down in the progress of life-expectancy related to the stabilization of a very low level of infant mortality (0.2–1% of births in the healthiest countries in the world) (Wilmoth, 2000). The present slow progress in high-income countries is mostly due to reduced mortality rates of chronic non-communicable diseases, principally among cardiovascular diseases and cancers. However, those advancements have a much lower impact on life-expectancy as compared to vaccination campaigns (Wilmoth, 2000; Olshansky et al., 2001; Buchanan, 2016). In these countries, the last two decades showed a slow-down and even a reduction of life-expectancy in specific populations, such as the Euro-American women in the USA, that may indicate the first change in life-expectancy trends (Case and Deaton, 2015; Shiels, 2017).

Predicting a continuous linear growth of life-expectancy in the long term may probably not be relevant if the major progresses have already been accomplished. Beyond the fittest mathematical model for estimating future trends, we need to carefully examine the consistency with structural and functional limits determining maximal lifespan related to life-history strategies and evolutionary and environmental constraints (Carnes and Olshansky, 2007; Hayflick, 2007; Olshansky and Carnes, 2009; Le Bourg, 2012; Le Bourg and Vijg, 2017; Vijg and Le Bourg, 2017). For example, aging is an irreversible process: it is complex as it concerns all physiological functions, organs, and maintenance systems. But, it also has universal characteristics, showing a continuous exponential decline starting in the third decade for all maximal indicators with an accelerated loss of physical performance until death (Moore, 1975; Berthelot et al., 2011; Marck et al., 2016). Despite the recent phenotypic expansion, such a widespread dynamic remained, showing no escape from decline, despite the best efforts of the oldest old (Moore, 1975; Berthelot et al., 2011; Marck et al., 2016).

**Figure 2:**
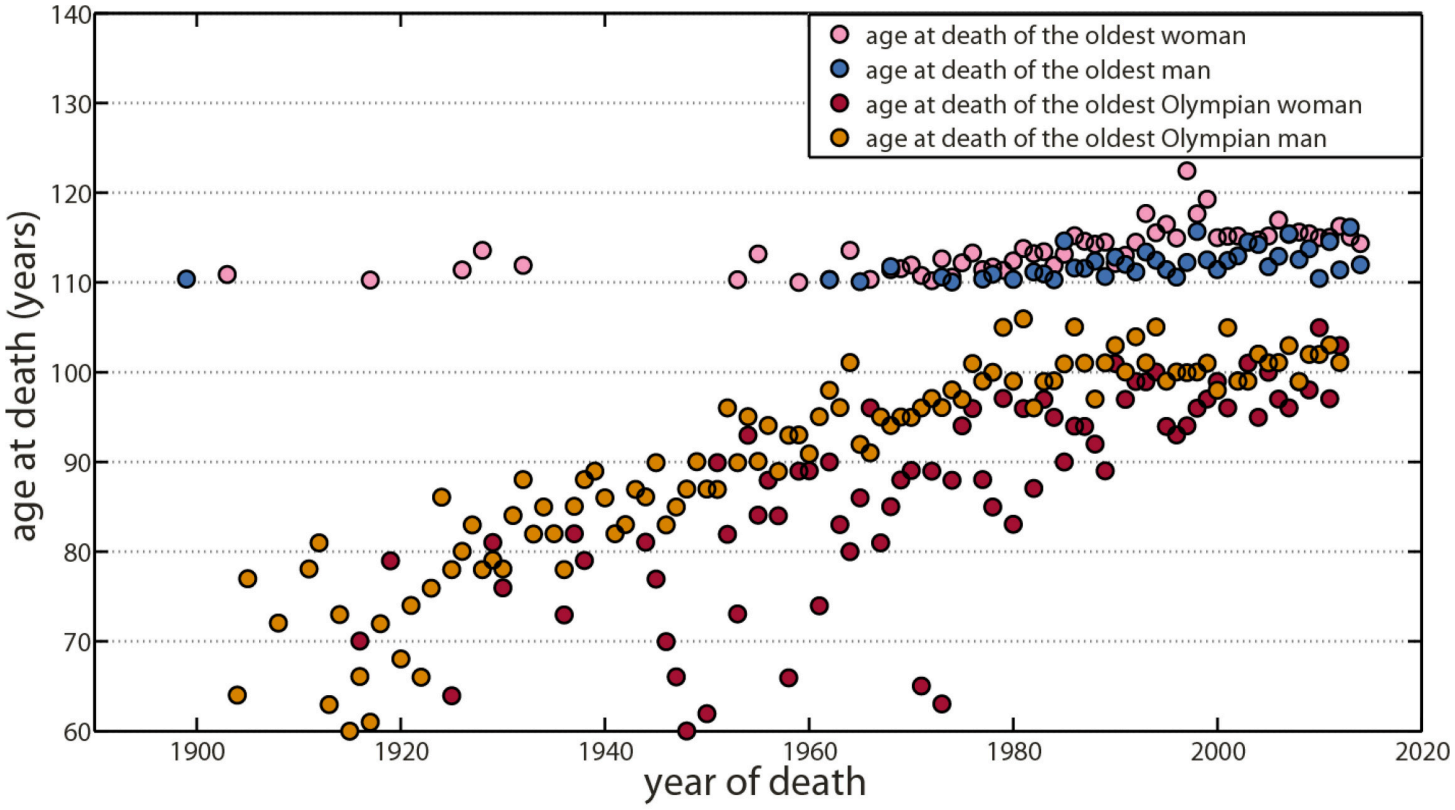
Maximal lifespan trends have shown a plateau for the oldest supercentenarian and the oldest Olympian. Since Jeanne Calment, who currently holds the lifespan record at 122.4 years in 1997, no one has lived more than 120 years and data suggest a plateau around 115–117 years that may indicate the potential biological upper limit of our species in terms of longevity. Oldest supercentenarian trends have shown a plateau for both women (purple) and men (blue). Similarly, maximal lifespan trends for oldest Olympians have shown a plateau for both women (red) and men (orange). Data for supercentenarians are available at the Gerontological Research Group (GRG; http://www.grg.org/). Data for Olympian athletes came from the most authoritative source of Olympian biographies (Clarke et al., 2012; Antero-Jacquemin et al., 2015).

Similarly, maximal lifespan increased slightly during the last two centuries (Robine and Vaupel, 2002), but since 1997, nobody has lived for more than 120 years. Surpassing mathematical models, projecting 300 years in the future without biological considerations, most recent data showed evidence of a lifespan plateau around 115–120 years (Le Bourg, 2012; da Silva Antero-Jacquemin et al., 2014; Dong et al., 2016), despite a sharp increase in the number of centenarians and supercentenarians (Figure 2). Jeanne Calment with 122.4 years has certainly come close to the potential biological limit of our species in term of longevity, at the benefit of an extremely rare long-lived phenotype supported by a specific lifestyle and chance (Dong et al., 2016).

Olympian and elite athletes also constitute a highly selected population with a 6–7 years longer life duration as compared to the general population (Marijon et al., 2013; Antero-Jacquemin et al., 2015). With a maximum of 106 years, maximal lifespan in this population paralleled the supercentenarian trend (da Silva Antero-Jacquemin et al., 2014). Both populations indicate a similar densification beyond the upper limit: more and more individuals reach values close to the limit, but do not surpass it, resulting in a progressive rectangularization of their survival curve (Kannisto, 2000; Andersen et al., 2012; da Silva Antero-Jacquemin et al., 2014).

### Adult height

Human height is another simple and accurate biomarker integrating both environmental and nutritional conditions encountered during fetal development and childhood (Fogel, 2004; Komlos and Baur, 2004; NCD-RisC, 2016; Stulp and Barrett, 2016). The recent study by the NCD Risk Factor Collaboration offers a large overview of secular trends in adult height for most countries in the world (NCD-RisC, 2016). The average gain in adult height for cohorts born between 1896 and 1996 was estimated at 8.3 ± 3.6 cm for women and 8.8 ± 3.5 cm for men (Larnkjær et al., 2006; Baten and Blum, 2012; Schönbeck et al., 2013; NCD-RisC, 2016; Stulp and Barrett, 2016).

**Figure 3:**
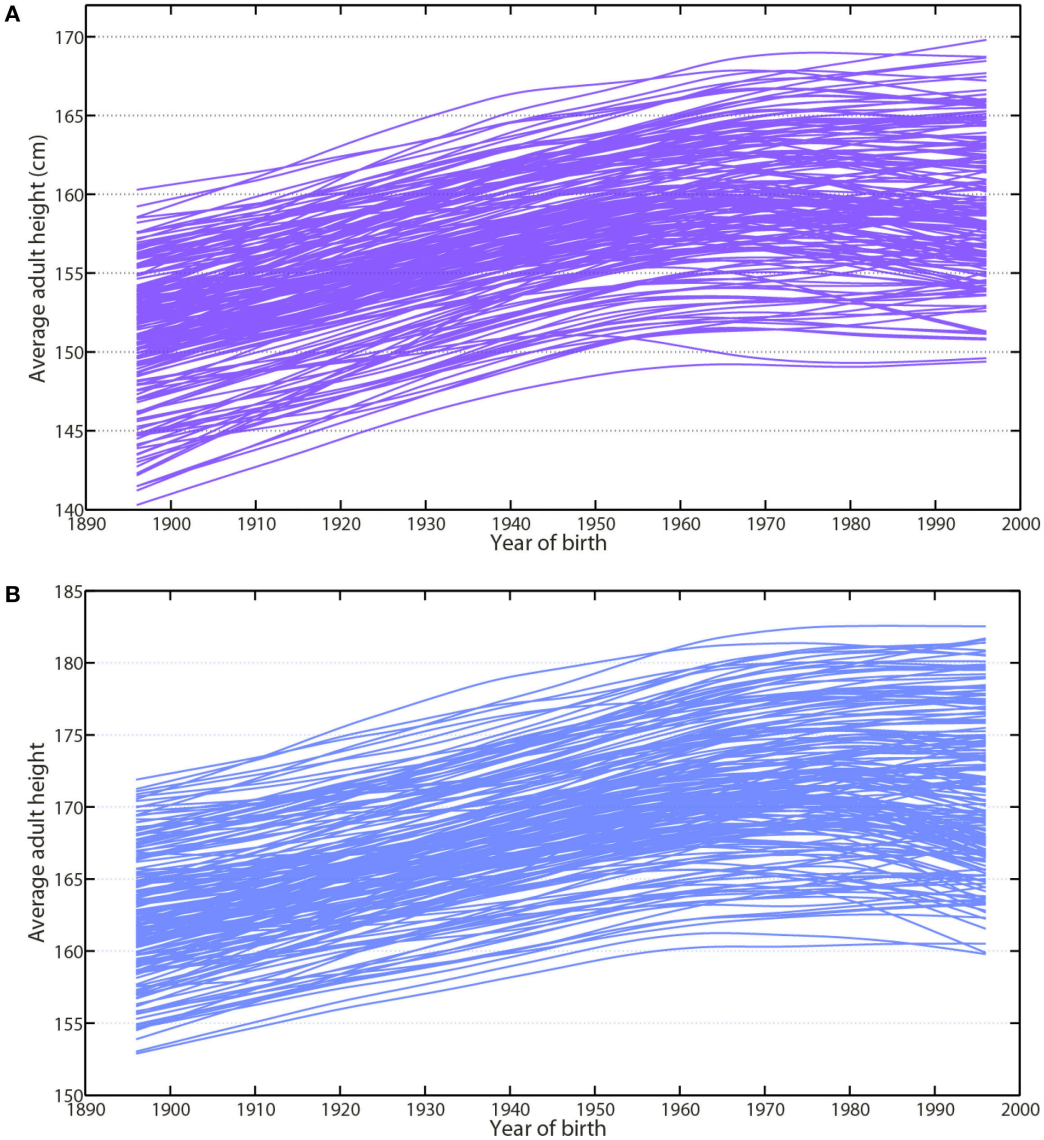
Average adult height has followed a similar pattern toward a plateauing trend since 1980. The analysis of the growth patterns reveals that the gain in height during the last century is not a linear process. During the last 3 decades, data have shown a similar plateauing state in the tallest populations among women and men of high-income countries from North America to Europe. This recent asymptote suggests a potential upper limit to human height. **(A)** Average adult height of women (violet-purple) represented for each country from 1914 up to 2014. **(B)** Average adult height of men (blue) represented for each country from 1914 up to 2014. Data are compiled from NCD-RisC and available by country on their website (https://elifesciences.org/content/5/e13410).

The analysis of growth patterns within world regions reveals that the gain in height during the last century was not a linear process (Komlos and Baur, 2004; Larnkjær et al., 2006; Komlos and Lauderdale, 2007; Rashad, 2008; Komlos, 2010; Baten and Blum, 2012; Schönbeck et al., 2013; NCD-RisC, 2016) (Figures 3A,B). During the last three decades, data have shown a similar plateau in the tallest populations among high-income countries from North America to Europe (Komlos and Baur, 2004; Larnkjær et al., 2006; Komlos and Lauderdale, 2007; Rashad, 2008; Komlos, 2010; Schönbeck et al., 2013; NCD-RisC, 2016; Stulp and Barrett, 2016) (Figures 3A,B). This recent asymptote suggests, we are approaching another of our upper limits (Le Bourg, 2012; NCD-RisC, 2016).

Such a trend was also observed among elite athletes (Norton and Olds, 2001; Sedeaud et al., 2014). After a large gain in the mid-twentieth century, a plateau has been described for all major US sports, which select especially tall individuals (Sedeaud et al., 2014). For example, in the NFL (National Football League) from 1920 to 2010, average players' height gained 8.1 cm (0.9 cm/decade, from 179.6 to 187.7 cm). However, during the last 30 years, male height has plateaued at 187 cm (Sedeaud et al., 2014). The NBA (National Basketball Association) evidenced the most obvious ceiling (Sedeaud et al., 2014): about 200 cm, since 1984. NHL, MLB (Major league Baseball), NFL and NBA height evolutions suggest a similar common trend linked to the best metric and chronometric performances among highly selected athletes (Sedeaud et al., 2014; Berthelot et al., 2015).

## What are the implications?

For millennia, it was difficult to test whether physiological limits do exist in humans, because no variation was accurately measurable. It is now possible to ascertain the biological limits of the human species, through sport records, lifespan, or height. These traits no longer increase, despite further continuous nutritional, medical, and scientific progress. This suggests that modern societies have allowed our species to reach its limits. We are the first generation to become aware of these limitations.

Regarding sport records, the first consequence is that increasingly less records will be broken in the coming years (Berthelot et al., 2008, 2010b). Humans need perspectives and, for this reason, in an attempt to reactivate interest in athletic progress, it was already proposed to start a new series of records or to change event rules (Spedding and Spedding, 2008; Berthelot et al., 2015). In this respect, the 2 h and 25 s marathon ran by Eliud Kipchoge for an exhibition in Italy has shown how improvement can be obtained under totally optimized and artificial conditions. Such improvement will substantially and artificially enhance maximal performance without reconsidering the general idea of existing upper biological limits.

In fact, it is probable that human natural limits have already been enhanced by artificial means for both maximal longevity and maximal physical performance. These “manufactured times”, represent an increase in life duration or sport performance, beyond the limits imposed by our biology (Carnes and Olshansky, 2007). In this sense, a scientific breakthrough may point to another future substantial “manufactured” gain that will shift the upper limits beyond the current values in addition to the healthy living standards that will increase the number of people reaching old age. However, such artificial enhancements will also have “Achilles heels”, i.e., maximal progress that cannot surpass an imposed ceiling. For example, the evolutionary constraints of body design that lead to structural and functional limitations or environmental factors hinder increased progress. The emergence of new major artificial enhancements may be less favorable in light of ever increasing environmental boundaries.

For such reasons, it is meaningless to claim that most human will live for 200–500 years in the near future (de Grey, 2003), thanks to medical or scientific progress, or that “within 15 years, we'll be adding more than a year every year to our remaining life expectancy” (Kurzweil and Grossman, 2010). Raising false hopes without taking into account that human beings are already extremely “optimized” for lifespan seems inappropriate.

In the scenario of limited performance, the interest on strategies for increasing quality of life reaches greater importance, such as investing in grassroots sports in order to enhance health (European Commission, 2016). If a country is able to promote human development and health, one should observe an incremental rise in values of mean adult height, sport performance, and lifespan. The utmost challenge is to maintain these indices at high values. Under escalating environmental constraints, this may cost increasingly more energy and investment to balance the rising ecosystemic pressures in order to maintain our performance levels. Indeed observing decreasing tendencies may provide an early signal that something has changed but not for the better. Human height has decreased in the last decade in some African countries; this suggests some societies are no longer able to provide sufficient nutrition for each of their children and maintain the health of their younger inhabitants (NCD-RisC, 2016).

Knowing limits of the human species indicates clear goals for any nation; states should govern in such a way that human size, lifespan, and physical performance increase in order to reach their highest values for most people. When plateaus are reached, care should then be taken to prevent regression even if remaining close to the upper limits may become more costly. This aim will be one of the most intense challenges of this century, especially with the new pressure of anthropocenic activities responsible for deleterious effects on both humans, environment and health (Rockström et al., 2009; Steffen et al., 2011, 2015; Carnes et al., 2014; Finch et al., 2014; McMichael, 2014). However, solutions may be found and collective actions may taken to restrain this pressure and, in an optimistic way, maintain the possibility of reaching and remaining at the upper limits(United Nations, 2016).

## Author contributions

AM, JA, GS, and GeB collected the data. AM, JA, GS, and GeB analyzed them. AM, JA, GeB, VMD, GiB, MS, ELB, and JFT wrote the paper and all authors contributed to the scientific debate. JFT is the guarantor.

### Conflict of interest statement

The authors declare that the research was conducted in the absence of any commercial or financial relationships that could be construed as a potential conflict of interest.
